# Contribution of alcohol use in HIV/hepatitis C virus co‐infection to all‐cause and cause‐specific mortality: A collaboration of cohort studies

**DOI:** 10.1111/jvh.13863

**Published:** 2023-06-20

**Authors:** Adam Trickey, Suzanne M. Ingle, Anders Boyd, M. John Gill, Sophie Grabar, Inma Jarrin, Niels Obel, Giota Touloumi, Robert Zangerle, Andri Rauch, Christopher T. Rentsch, Derek D. Satre, Michael J. Silverberg, Fabrice Bonnet, Jodie Guest, Greer Burkholder, Heidi Crane, Ramon Teira, Juan Berenguer, Christoph Wyen, Sophie Abgrall, Mojgan Hessamfar, Peter Reiss, Antonella d’Arminio Monforte, Kathleen A. McGinnis, Jonathan A. C. Sterne, Linda Wittkop, the Antiretroviral Therapy Cohort Collaboration

**Affiliations:** ^1^ Population Health Sciences University of Bristol Bristol UK; ^2^ Stichting HIV Monitoring Amsterdam The Netherlands; ^3^ Department of Infectious Diseases Public Health Service of Amsterdam Amsterdam The Netherlands; ^4^ Amsterdam UMC University of Amsterdam, Infectious Diseases Amsterdam The Netherlands; ^5^ South Alberta HIV Clinic, Department of Medicine University of Calgary Calgary Canada; ^6^ Sorbonne Université, INSERM, Institut Pierre Louis d'Épidémiologie et de Santé Publique (IPLESP) Paris France; ^7^ Department of Public Health AP‐HP, St Antoine Hospital Paris France; ^8^ National Centre of Epidemiology Carlos III Health Institute Madrid Spain; ^9^ CIBER de Enfermedades Infecciosas Instituto de Salud Carlos III; ^10^ Department of Infectious Diseases Copenhagen University Hospital, Rigshospitalet Copenhagen Denmark; ^11^ Department of Hygiene, Epidemiology and Medical Statistics, Medical School National and Kapodistrian University of Athens Athens Greece; ^12^ Austrian HIV Cohort Study (AHIVCOS) Medizinische Universität Innsbruck Innsbruch Austria; ^13^ Department of Infectious Diseases, Inselspital Bern University Hospital, University of Bern Bern Switzerland; ^14^ Yale School of Medicine and VA Connecticut Healthcare System West Haven Connecticut USA; ^15^ Faculty of Epidemiology and Population Health London School of Hygiene and Tropical Medicine London UK; ^16^ Department of Psychiatry and Behavioral Sciences Weill Institute for Neurosciences, University of California San Francisco USA; ^17^ Division of Research Kaiser Permanente Northern California Oakland California USA; ^18^ Institut Bergonié, BPH, U1219, CIC‐EC 1401, INSERM, Univ. Bordeaux Bordeaux France; ^19^ CHU de Bordeaux, Service de Médecine Interne et Maladies Infectieuses, INSERM Institut Bergonié Hôpital St‐André, CIC‐EC 1401 Bordeaux France; ^20^ Atlanta VA Medical Center Decatur Georgia USA; ^21^ Rollins School of Public Health at Emory University Atlanta Georgia USA; ^22^ University of Alabama Birmingham Alabama USA; ^23^ Department of Medicine University of Washington Seattle Washington USA; ^24^ Servicio de Medicina Interna Hospital Universitario de Sierrallana Torrelavega Spain; ^25^ Hospital General Universitario Gregorio Marañón Madrid Spain; ^26^ Department I for Internal Medicine University Hospital of Cologne Cologne Germany; ^27^ APHP, Service de Médecine Interne, Hôpital Béclère Clamart France; ^28^ CESP, INSERM U1018, Université Paris‐Saclay, UVSQ, Le Kremlin‐Bicêtre Villejuif France; ^29^ Department of Global Health Amsterdam University Medical Centers Amsterdam The Netherlands; ^30^ Amsterdam Institute for Global Health and Development Amsterdam The Netherlands; ^31^ Clinic of Infectious and Tropical Diseases, Department of Health Sciences ASST Santi Paolo e Carlo, University Hospital Milan Italy; ^32^ NIHR Bristol Biomedical Research Centre Bristol UK; ^33^ Health Data Research UK South‐West Bristol UK; ^34^ INRIA SISTM Team Talence France; ^35^ CHU de Bordeaux, Service d'information Médicale, INSERM Institut Bergonié, CIC‐EC 1401 Bordeaux France

**Keywords:** alcohol, cause‐specific, cohort, hepatitis C virus, HIV, mortality

## Abstract

Among persons with HIV (PWH), higher alcohol use and having hepatitis C virus (HCV) are separately associated with increased morbidity and mortality. We investigated whether the association between alcohol use and mortality among PWH is modified by HCV. Data were combined from European and North American cohorts of adult PWH who started antiretroviral therapy (ART). Self‐reported alcohol use data, collected in diverse ways between cohorts, were converted to grams/day. Eligible PWH started ART during 2001–2017 and were followed from ART initiation for mortality. Interactions between the associations of baseline alcohol use (0, 0.1–20.0, >20.0 g/day) and HCV status were assessed using multivariable Cox models. Of 58,769 PWH, 29,711 (51%), 23,974 (41%) and 5084 (9%) self‐reported alcohol use of 0 g/day, 0.1–20.0 g/day, and > 20.0 g/day, respectively, and 4799 (8%) had HCV at baseline. There were 844 deaths in 37,729 person‐years and 2755 deaths in 443,121 person‐years among those with and without HCV, respectively. Among PWH without HCV, adjusted hazard ratios (aHRs) for mortality were 1.18 (95% CI: 1.08–1.29) for 0.0 g/day and 1.84 (1.62–2.09) for >20.0 g/day compared with 0.1–20.0 g/day. This J‐shaped pattern was absent among those with HCV: aHRs were 1.00 (0.86–1.17) for 0.0 g/day and 1.64 (1.33–2.02) for >20.0 g/day compared with 0.1–20.0 g/day (interaction *p* < .001). Among PWH without HCV, mortality was higher in both non‐drinkers and heavy drinkers compared with moderate alcohol drinkers. Among those with HCV, mortality was higher in heavy drinkers but not non‐drinkers, potentially due to differing reasons for not drinking (e.g. illness) between those with and without HCV.

**Abbreviations**
aHR: adjusted hazard ratio
AIDS: autoimmune deficiency disorder
ART: antiretroviral therapy
ART‐CC: Antiretroviral Therapy Cohort Collaboration
AUDIT‐C: Alcohol Use Disorders Identification Test Consumption
CI: confidence interval
DAA: direct acting antiviral
HBsAg: hepatitis B surface antigen
HBV: hepatitis B virus
HCV: hepatitis C virus
HR: hazard ratio
IDU: injecting drug use
PWH: persons with HIV
RNA: ribonucleic acid

## 1 INTRODUCTION

Mortality is higher among persons with HIV (PWH) who also have hepatitis C virus (HCV) than those who do not.^1^, ^2^ This is despite early administration of combination antiretroviral therapy (ART) and durable suppression of HIV replication having improved overall survival and delayed disease progression among PWH.^3^ In some settings, liver‐related mortality has been ranked as a leading cause of mortality among PWH,^4^, ^5^, ^6^, ^7^ likely due to hepatic decompensation and/or hepatocellular carcinoma.^8^, ^9^, ^10^

Since the advent of direct acting antiviral (DAA)‐based treatment in 2014, sustained virological response (SVR) rates are >90% in HIV/HCV co‐infected patients,^11^, ^12^, ^13^ who were on the priority list for DAA initiation soon after their arrival on the market. DAA treatment is now recommended for all persons with HCV.^14^ Lifestyle factors, especially risk behaviours, are likely to differ between PWH with and without HCV; the prevalence of HCV among PWH with histories of injecting drug use (IDU) is estimated to be 82%.^15^ Furthermore, HCV is frequent (weighted average 16.3%) among patients with alcohol use disorders.^16^ Alcohol use in PWH with HCV has been related to increased risk of liver disease progression (liver fibrosis, liver cancer and liver‐related mortality),^17^, ^18^ and no safe level of alcohol use has been described. Excess mortality in PWH with HCV could be addressed by interventions to reduce harm from substance/alcohol use and other risk behaviours. However, data on the association of alcohol use with all‐cause and cause‐specific mortality in PWH with HCV are scarce, especially in the era of DAAs.

Studies of alcohol use among PWH have found J‐ or U‐shaped associations with mortality: those with no alcohol use and heavy alcohol use have higher mortality than those with low/moderate alcohol use.^19^ Higher mortality among non‐drinkers may arise because some members of this group stopped drinking due to illness or alcohol dependency.^20^, ^21^ Whether the same patterns holds among PWH with HCV is unclear.

We aimed to investigate whether the association between alcohol use and all‐cause mortality in PWH differed by HCV status, and whether any such difference remained after accounting for HCV cure. We then investigated these outcomes in separate follow‐up periods to account for the availability of DAAs and assessed trends in all‐cause and cause‐specific mortality in groups of PWH defined by HCV and alcohol use.

## 2 MATERIALS AND METHODS

The Antiretroviral Therapy Cohort Collaboration (ART‐CC) combines data from cohorts of PWH in Europe and North America. Ethics committees or institutional review boards approved the individual cohorts, which used standardised data collection methods and regularly followed‐up patients. The 14 cohorts included in these analyses were those with data on both grams of alcohol use per day (recorded from 6 months before starting ART) and baseline HCV RNA testing data available for over 50 patients. Included PWH started ART between 2001 and 2017 when aged ≥16 years old,^22^ and had a CD4 count and HIV‐1 RNA viral load measurement between 3 months before and 2 weeks after starting their first combination ART regimen. We excluded those with known hepatitis B virus (HBV) infection, defined as testing positive for hepatitis B surface antigen (HBsAg) at baseline: data on HBV testing were unavailable for one cohort that was included.

### 2.1 Alcohol data

Data on self‐reported alcohol use were recorded through questionnaires that varied between cohorts and included both alcohol use disorders identification test consumption (AUDIT‐C) and non‐AUDIT‐C measures. AUDIT‐C is a three‐item questionnaire which returns a score from 0 to 12 where increasing score means higher risk alcohol use. Non‐AUDIT‐C cohorts recorded the self‐reported number of drinks/units per week/day. These data were harmonised into grams of alcohol per day taking the mid‐points of categories for cohorts that measured alcohol use in categories (see supplementary materials for further information). Distributions of alcohol use within cohorts were examined, following which they were categorised as 0 , 0.1–20.0 and > 20.0 g per day, to enable analyses to be combined across cohorts, while still accounting for no alcohol use, low to medium alcohol use and high alcohol use. This alcohol use measure was taken at a single time point closest to ART start for each person, using a window of 6 months prior to ART start until the end of follow‐up. In a sensitivity analysis, we restricted the sample to those with alcohol measurements taken from 6 months before ART initiation up to 3 months afterwards.

### 2.2 HCV data

HCV‐RNA status at baseline was used to define baseline HCV status when available (for 47% of patients with HCV), otherwise HCV antibody status was used. Among PWH with HCV antibody status at baseline but no baseline HCV RNA data, data on HCV‐RNA testing were subsequently available for 59%, and the earliest test was positive for 86%. Time‐updated HCV status after baseline was defined using only HCV RNA status. HCV cure or spontaneous clearance was defined as two subsequent negative HCV RNA tests following a positive test, while new HCV infection was defined as a positive HCV RNA test following a negative test. Data on HCV treatment were incomplete, and so were not used in analyses.

### 2.3 Mortality data

Cohorts gathered information on mortality through linkage with vital statistics agencies and hospitals or physician report, and the active follow‐up of participants. We adapted the Coding of Death in HIV (CoDe) project protocol (https://www.chip.dk/Tools‐Standards/CoDe/About) to classify causes of death, as described previously,^23^ with causes of death further categorised into groups and tabulated against HCV/alcohol use status.

### 2.4 Statistical analyses

Patients were followed‐up from ART start (baseline) until the earliest of death, loss to follow‐up or cohort‐specific database administrative censoring (mostly in 2019). Patients with a gap of greater than 1 year between the date last known to be alive and administrative censoring were considered lost to follow‐up and censored 6 months after their last recorded measurement.

We fitted Cox models with baseline hazards stratified by cohort to estimate associations of alcohol use with all‐cause mortality according to HCV status (negative, positive) by including interaction terms. Follow‐up from 2001 to 2017 was split at times of HCV cure, spontaneous clearance or infection (including reinfection), allowing for multiple periods of follow‐up per person. We then further stratified follow‐up time into calendar periods 2001–2013 and 2014–2017, to account for the availability of DAAs from 2014 onwards. The covariates included were self‐reported HIV acquisition mode (sex between men, IDU, heterosexual sex, other/unknown), sex (male or female) and variables time‐updated at times of HCV cure, clearance, or infection: prior AIDS events (binary), as well as age (years), CD4 count (cells/μL) and log HIV‐1 RNA (copies/mL). CD4 count and log HIV‐1 RNA values were modelled using cubic splines with three knots and time‐updated measurements were taken from windows between 6 months before and 1 month after the time of cure or infection. We investigated violations of the proportional hazards assumption using Schoenfeld residuals: these suggested a time varying effect of prior AIDS events so interactions between prior AIDS events and time (0–6, 6–12, 12–24, >24 months) were included in subsequent models.

In additional analyses we stratified HCV negative PWH into those who had never been HCV positive and those who were HCV negative following cure or spontaneous clearance. Finally, using follow‐up after 2014 among PWH who had ever had HCV, we compared mortality rates for different alcohol use categories separately among PWH who were previously HCV‐positive and PWH who were HCV‐positive at the time.

## 3 RESULTS

There were 480,849 person‐years follow‐up (median 7.8 years) among 58,769 PWH, of whom 12,454 (21%) were female. At ART initiation, the median age was 40 years (interquartile range: 32–49), while 4799 (8%) had HCV. The daily alcohol use was 0 g in 29,711 (51%) PWH, 0.1–20.0 g in 23,974 (41%), and > 20.0 g in 5084 (9%). The median timing of the measurement of daily alcohol use was taken 2 months after ART start (interquartile range: 0 months before to 3 years after ART start). Table 1 presents the characteristics of those included stratified by HCV status and alcohol use category. A higher percentage of persons that acquired HIV through IDU were in the > 20.0 g alcohol use category (20%), than among those that acquired HIV sexually (9%), or through other/unknown modes of acquisition (6%).

**TABLE 1:** Characteristics at start of ART stratified by HCV status and daily alcohol use category.

	Total	HCV negative			HCV positive		
		0 g	0.1‐20 g	>20 g	0 g	0.1‐20 g	>20 g
Overall	58,769 (100%)	27,202 (50%)	22,336 (41%)	4432 (8%)	2509 (52%)	1638 (34%)	652 (14%)
Age (years)							
16–29	10,705 (18%)	5555 (20%)	4163 (19%)	667 (15%)	152 (6%)	116 (7%)	52 (8%)
30–39	17,921 (30%)	8693 (32%)	6994 (31%)	1178 (27%)	561 (22%)	321 (20%)	174 (27%)
40–49	16,502 (28%)	7136 (26%)	6228 (28%)	1390 (31%)	916 (37%)	575 (35%)	257 (39%)
≥50	13,641 (23%)	5818 (21%)	4951 (22%)	1197 (27%)	880 (35%)	626 (38%)	169 (26%)
Sex							
Male	46,315 (79%)	18,939 (70%)	19,362 (87%)	4089 (92%)	1953 (78%)	1411 (86%)	561 (86%)
Female	12,454 (21%)	8263 (30%)	2974 (13%)	343 (8%)	556 (22%)	227 (14%)	91 (14%)
ART start year							
2001–2013	46,174 (79%)	21,036 (77%)	17,615 (79%)	3322 (75%)	2191 (87%)	1462 (89%)	548 (84%)
2014–2017	12,595 (21%)	6166 (23%)	4721 (21%)	1110 (25%)	318 (13%)	176 (11%)	104 (16%)
CD4 count (cells/μL)							
0–49	7037 (12%)	3643 (13%)	2304 (10%)	512 (12%)	324 (13%)	185 (11%)	60 (9%)
50–99	4363 (7%)	2184 (8%)	1452 (7%)	331 (7%)	187 (7%)	144 (9%)	65 (10%)
100–199	9339 (16%)	4305 (16%)	3383 (15%)	690 (16%)	483 (19%)	332 (20%)	146 (22%)
200–349	18,048 (31%)	8118 (30%)	6971 (31%)	1389 (31%)	804 (32%)	532 (32%)	234 (36%)
≥350	19,982 (34%)	8952 (33%)	8226 (37%)	1501 (34%)	711 (28%)	445 (27%)	147 (23%)
Viral load (copies/mL)							
0–9999	9881 (17%)	4581 (17%)	3705 (17%)	597 (13%)	536 (21%)	347 (21%)	115 (18%)
10,000–99,999	23,287 (40%)	10,640 (39%)	8944 (40%)	1768 (40%)	1019 (41%)	644 (39%)	272 (42%)
≥100,000	25,601 (44%)	11,981 (44%)	9687 (43%)	2067 (47%)	954 (38%)	647 (39%)	265 (41%)
HIV acquisition mode							
Sex between men	22,576 (38%)	9406 (35%)	10,389 (47%)	2286 (52%)	231 (9%)	205 (13%)	59 (9%)
IDU	2502 (4%)	377 (1%)	342 (2%)	156 (4%)	853 (34%)	433 (26%)	341 (52%)
Heterosexual sex	20,487 (35%)	12,463 (46%)	5926 (27%)	1348 (30%)	440 (18%)	212 (13%)	98 (15%)
Other/unknown	13,204 (22%)	4956 (18%)	5679 (25%)	642 (14%)	985 (39%)	788 (48%)	154 (24%)
AIDS status							
No	47,385 (81%)	21,872 (80%)	18,382 (82%)	3488 (79%)	1906 (76%)	1253 (76%)	484 (74%)
Yes	11,384 (19%)	5330 (20%)	3954 (18%)	944 (21%)	603 (24%)	385 (24%)	168 (26%)
HCV status							
Negative	53,970 (92%)	27,202 (100%)	22,336 (100%)	4432 (100%)	0 (0%)	0 (0%)	0 (0%)
Positive	4799 (8%)	0 (0%)	0 (0%)	0 (0%)	2509 (100%)	1638 (100%)	652 (100%)

Abbreviations: ART, antiretroviral therapy; HCV, hepatitis C virus; IDU, injecting drug use.

### 3.1 All‐cause mortality

Of 3599 deaths (overall mortality rate 7.5 per 1000 person‐years), 844 in 37,729 person‐years follow‐up were in PWH with HCV and 2755 in 443,121 person‐years in PWH without HCV. The numbers of deaths (person‐years) were 1789 (244,405) among those reporting no alcohol use, 1302 (196,642) among those reporting alcohol use of 0.1 g–20 g/day, and 508 deaths in 39,802 follow‐up years and >20 g per day, respectively.

### 3.2 Mortality hazard ratios

Table 2 shows adjusted mortality hazard ratios (aHRs) comparing alcohol use categories, stratified by HCV status and year of ART initiation; unadjusted HRs are shown in Table S1. Overall, there was a J‐shaped pattern, with aHRs of 1.17 (95% confidence interval [95% CI]: 1.09–1.27) for those with alcohol use of 0.0 g/day and 1.88 (95% CI: 1.68–2.09) with alcohol use of >20.0 g/day, compared with 0.1–20.0 g/day. A similar pattern was seen in those without HCV, with aHRs 1.18 (95% CI: 1.08–1.29) for 0.0 g/day and 1.84 (95% CI: 1.62–2.09) for >20.0 g/day, compared with 0.1–20.0 g/day. However, these associations differed in those with HCV (interaction *p*‐value<0.001): aHRs were 1.00 (95% CI: 0.86–1.17) for 0.0 g/day and 1.64 (95% CI: 1.33–2.02) for >20.0 g/day, compared with 0.1–20.0 g/day. These associations were similar when based on follow‐up between 2001–2013 and 2014–2017, although for PWH with HCV the aHR for >20.0 g/day of alcohol use, compared with 0.1–20.0 g/day was attenuated to 1.47 (95% CI: 1.07–2.03) during 2014–17. For PWH reporting 0.1–20.0 g/day of alcohol, the aHR was 2.64 (95% CI: 2.28–3.04) comparing those with and without HCV. In a sensitivity analysis restricting the sample to 30,208 PWH with alcohol use values taken between 6 months before ART initiation to 3 months afterwards, results were similar to in the main analysis: aHRs 1.12 (95% CI: 0.99–1.26) for 0.0 g/day and 1.69 (95% CI: 1.44–1.97) for >20.0 g/day, compared with 0.1–20.0 g/day.

**TABLE 2:** Adjusted^a^ mortality hazard ratios for alcohol use categories, stratified by calendar year period and HCV status (negative, positive), splitting follow‐up time by HCV status.

	All patients		Without HCV		With HCV		
Alcohol use (g/day)	Mortality rate per 1000 person‐years (95% CI)	aHR (95% CI)	Mortality rate per 1000 person‐years (95% CI)	aHR (95% CI)	Mortality rate per 1000 person‐years (95% CI)	aHR (95% CI)	Interaction *p*‐value
Follow‐up between 2001 and 2017 (*N* = 58,769)							
0.0 g	7.3 (7.0–7.7)	1.17 (1.09–1.27)	6.1 (5.8–6.4)	1.18 (1.08–1.29)	21.5 (19.5–23.7)	1.00 (0.86–1.17)	
0.1–20.0 g	6.6 (6.3–7.0)	1	5.5 (5.2–5.9)	1	21.4 (19.0–24.0)	1	<.001
>20.0 g	12.8 (11.8–13.9)	1.88 (1.68–2.09)	10.5 (9.5–10.7)	1.84 (1.62–2.09)	28.6 (24.2–33.7)	1.64 (1.33–2.02)	
Follow‐up between 2001 and 2013 (*N* = 46,174)							
0.0 g	6.4 (5.9–6.8)	1.18 (1.05–1.32)	5.0 (4.6–5.4)	1.20 (1.05–1.37)	19.2 (17.0–21.8)	0.97 (0.79–1.18)	
0.1–20.0 g	5.8 (5.3–6.2)	1	4.5 (4.1–4.9)	1	19.4 (16.7–22.5)	1	<.001
>20.0 g	11.8 (10.4–13.3)	1.98 (1.69–2.33)	9.2 (7.9–10.7)	1.94 (1.61–2.35)	26.3 (21.2–32.6)	1.70 (1.29–2.23)	
Follow‐up between 2014 and 2017 (*N* = 54,884)							
0.0 g	8.4 (7.9–9.0)	1.17 (1.06–1.30)	7.3 (6.9–7.9)	1.18 (1.05–1.32)	26.1 (22.4–30.3)	1.04 (0.82–1.32)	
0.1–20.0 g	7.6 (7.0–8.1)	1	6.6 (6.1–7.2)	1	25.0 (20.9–29.9)	1	<.001
>20.0 g	13.9 (12.3–15.7)	1.76 (1.51–2.05)	12.0 (10.4–13.7)	1.75 (1.48–2.07)	32.7 (25.2–42.4)	1.47 (1.07–2.03)	

Abbreviation: HCV, hepatitis C virus.

^a^ The covariates included were HIV acquisition group, female, prior AIDS status, age, CD4 count cells/μL and log HIV‐1 RNA copies/mL.

### 3.3 Mortality hazard ratios including time‐dependent HCV cure or clearance

Table 3 shows adjusted mortality HRs comparing alcohol use categories when additionally stratifying HCV status into periods following a cure or spontaneous clearance. Among PWH without HCV a J‐shaped pattern was seen overall and in the 2001–2013 and 2014–2017 time periods. The numbers of deaths (person years of follow‐up) were 844 (37,729) among those HCV positive and 103 (9846) among those whose HCV was cured/cleared. The small number of deaths among those cured/cleared meant that associations with alcohol consumption were imprecisely estimated in the 2001–2013 period. However, in the overall and 2014–2017 periods mortality was higher among PWH reporting >20.0 g compared with 0.1–20.0 g alcohol use, aHRs 1.87 (95% CI: 1.00–3.48) and 2.45 (95% CI: 1.24–4.82), respectively.

**TABLE 3:** Adjusted^a^ mortality hazard ratios (aHRs) for alcohol use category, stratified by calendar year period and HCV status (negative, positive, cured/cleared), splitting follow‐up time by HCV status.

Alcohol use (grams per day)	HCV negative		HCV positive		Cured/cleared	
	Mortality rate per 1000 person‐years (95% CI)	aHR (95% CI)	Mortality rate per 1000 person‐years (95% CI)	aHR (95% CI)	Mortality rate per 1000 person‐years (95% CI)	aHR (95% CI)
Follow‐up between 2001 and 2017 (*N* = 58,769) (interaction *p*‐value <0.001)						
0.0 g	6.0 (5.7–6.3)	1.18 (1.08–1.29)	21.5 (19.5–23.7)	1.00 (0.86–1.17)	11.0 (8.4–14.4)	1.13 (0.74–1.72)
0.1–20.0 g	5.5 (5.1–5.8)	1	21.4 (19.0–24.0)	1	8.7 (6.2–12.1)	1
>20.0 g	10.4 (9.4–11.6)	1.84 (1.62–2.09)	28.6 (24.2–33.7)	1.64 (1.33–2.02)	15.2 (9.0–25.7)	1.87 (1.00–3.48)
Follow‐up between 2001 and 2013 (*N* = 28,148) (interaction *p*‐value <0.001)						
0.0 g	4.9 (4.5–5.3)	1.21 (1.06–1.38)	19.2 (17.0–21.8)	0.97 (0.79–1.18)	7.3 (3.9–13.5)	0.58 (0.25–1.37)
0.1–20.0 g	4.4 (4.0–4.9)	1	19.4 (16.7–22.5)	1	9.9 (5.5–18.0)	1
>20.0 g	9.3 (8.0–10.8)	1.99 (1.64–2.40)	26.3 (21.2–32.6)	1.70 (1.29–2.23)	3.9 (0.6–27.8)	0.37 (0.05–2.87)
Follow‐up between 2014 and 2017 (*N* = 12,595) (interaction *p*‐value <0.001)						
0.0 g	7.2 (6.7–7.7)	1.17 (1.04–1.32)	26.1 (22.4–30.3)	1.04 (0.82–1.32)	12.5 (9.3–16.8)	1.35 (0.82–2.22)
0.1–20.0 g	6.6 (6.1–7.2)	1	25.0 (20.9–29.9)	1	8.2 (5.5–12.3)	1
>20.0 g	11.6 (10.1–13.4)	1.72 (1.45–2.04)	32.7 (25.5–42.4)	1.47 (1.07–2.03)	19.6 (11.4–33.7)	2.45 (1.24–4.82)

Abbreviation: HCV, hepatitis C virus.

^a^ The covariates included were HIV acquisition group, female, prior AIDS status, age, CD4 count cells/μL and log HIV‐1 RNA copies/mL.

### 3.4 Mortality hazard ratios among those who had ever had HCV

Table S2 shows aHRs comparing alcohol use categories in post‐2014 follow‐up among PWH who had ever had HCV, with follow‐up split into time HCV positive or after HCV cure or spontaneous clearance (4576 PWH contributed 8973 time‐periods). The overall aHR for mortality comparing >20.0 g with 0.1–20.0 g daily alcohol use was 1.32 (95% CI: 0.93–1.88). The mortality aHR was lower during follow‐up for PWH who were currently HCV‐positive (1.13 [95% CI: 0.76–1.69]), than for PWH who were previously HCV‐positive (2.32 [95% CI: 1.16–4.63]). The aHRs for mortality comparing 0.0 g with 0.1–20.0 g of daily alcohol use were 0.98 (95% CI: 0.74–1.29) and 1.51 (95% CI: 0.91–2.51) during follow‐up for PWH who currently HCV‐positive and previously HCV‐positive, respectively.

### 3.5 Cause‐specific mortality

Causes of death, stratified by alcohol use category and baseline HCV status are presented in Table 4. The proportion of liver‐related deaths among PWH who had HCV was higher (18.0%) than among those without HCV (3.1%). Most liver‐related deaths among those with HCV were due to hepatitis (including hepatitis‐related liver cancers), while most among PWH without HCV were due to liver failure. There was a higher proportion of liver‐related deaths among PWH with >20.0 g/day of alcohol use (10.0%), than among those with 0.1–20.0 g/day use (6.2%), or 0 g/day use (6.1%). The highest proportion of deaths due to substance use (5.1%) was among PWH with HCV who reported >20.0 g/day of alcohol use.

**TABLE 4:** Causes of death stratified by baseline alcohol use category and HCV RNA status.

Cause of death	All	HCV negative (*N* = 2723)			HCV positive (*N* = 876)		
	(*N* = 3599) MR: 7.5 [7.2–7.7]	0 g alcohol (*N* = 1356) MR: 6.1 [5.7–6.4]	0.1–20.0 g alcohol (*N* = 1003) MR: 5.5 [5.2–5.8]	>20.0 g alcohol (*N* = 364) MR: 10.5 [9.5–11.6]	0 g alcohol (*N* = 433) MR: 20.4 [18.6–22.4]	0.1–20.0 g alcohol (*N* = 299) MR: 21.1 [18.8–23.6])	>20.0 g alcohol (*N* = 144) MR: 28.0 [23.7–32.9]
Liver	242 (6.7%)	34 (2.5%)	23 (2.3%)	27 (7.4%)	76 (17.6%)	58 (19.4%)	24 (16.7%)
Unspecified viral hepatitis	10 (0.3%)	0 (0%)	0 (0%)	0 (0%)	5 (1.2%)	2 (0.7%)	3 (2.1%)
HCV	131 (3.6%)	3 (0.2%)	2 (0.2%)	3 (0.8%)	59 (13.6%)	47 (15.7%)	17 (11.8%)
HBV	2 (0.1%)	1 (0.1%)	1 (0.1%)	0 (0%)	0 (0%)	0 (0%)	0 (0%)
Liver failure	68 (1.9%)	23 (1.7%)	15 (1.5%)	23 (6.3%)	3 (0.7%)	3 (1%)	1 (0.7%)
Liver cancer	31 (0.9%)	7 (0.5%)	5 (0.5%)	1 (0.3%)	9 (2.1%)	6 (2%)	3 (2.1%)
Substance use	97 (2.7%)	20 (1.5%)	26 (2.6%)	15 (4.1%)	14 (3.2%)	13 (4.3%)	9 (6.3%)
Chronic alcohol use	19 (0.5%)	2 (0.1%)	6 (0.6%)	7 (1.9%)	1 (0.2%)	1 (0.3%)	2 (1.4%)
Other/unspecified	78 (2.2%)	18 (1.3%)	20 (2%)	8 (2.2%)	13 (3%)	12 (4%)	7 (4.9%)
AIDS	815 (22.6%)	344 (25.4%)	234 (23.3%)	66 (18.1%)	86 (19.9%)	58 (19.4%)	27 (18.8%)
CNS‐related	49 (1.4%)	28 (2.1%)	9 (0.9%)	3 (0.8%)	3 (0.7%)	2 (0.7%)	4 (2.8%)
Heart/vascular	200 (5.6%)	80 (5.9%)	64 (6.4%)	15 (4.1%)	26 (6%)	12 (4%)	3 (2.1%)
Cardiovascular	204 (5.7%)	94 (6.9%)	67 (6.7%)	16 (4.4%)	15 (3.5%)	8 (2.7%)	4 (2.8%)
Non‐AIDS infection	235 (6.5%)	98 (7.2%)	57 (5.7%)	20 (5.5%)	26 (6%)	23 (7.7%)	11 (7.6%)
Non‐AIDS, non‐hepatitis, non‐liver malignancy	668 (18.6%)	277 (20.4%)	214 (21.3%)	74 (20.3%)	47 (10.9%)	36 (12%)	20 (13.9%)
Respiratory	121 (3.4%)	49 (3.6%)	34 (3.4%)	12 (3.3%)	16 (3.7%)	10 (3.3%)	0 (0%)
Unnatural	178 (4.9%)	55 (4.1%)	60 (6%)	29 (8%)	14 (3.2%)	18 (6%)	2 (1.4%)
Other	222 (6.2%)	83 (6.1%)	51 (5.1%)	23 (6.3%)	31 (7.2%)	16 (5.4%)	18 (12.5%)
Unknown	568 (15.8%)	194 (14.3%)	164 (16.4%)	64 (17.6%)	79 (18.2%)	45 (15.1%)	22 (15.3%)

Abbreviations: CNS, central nervous system; HBV, hepatitis B virus; HCV, hepatitis C virus; MR, mortality rate per 1000 person‐years.

Among PWH who had HCV at baseline and reported >20.0 g/day of alcohol use, 16.7% of deaths were due to liver and 6.3% due to substance use‐related causes. The corresponding proportions among those with HCV were 19.4% and 4.3% among those with 0.1–20.0 g use per day, and 17.6% and 3.2% among those with 0 g use per day (Table 4). Among those without HCV, the corresponding proportions were 7.4% and 4.1%, 2.3% and 2.6%, and 2.5% and 1.5% among those who in the >20.0 , 0.1–20.0 , and 0 g/day alcohol use categories, respectively.

## 4 DISCUSSION

The association between alcohol use and all‐cause mortality in PWH is modified by HCV status. Among PWH without HCV, there was a J‐shaped pattern of higher mortality in those reporting no drinking and heavy drinking, compared with low or moderate drinking, while among PWH with HCV, there was higher mortality only among those reporting heavy drinking. Due to wide confidence intervals, further evidence is required to ascertain whether a J‐shaped pattern was present among PWH who had been cured of or cleared HCV. 22% of the deaths among PWH with HCV were liver‐ or substance use‐related, compared with only 5% among PWH without HCV. Among PWH without HCV, the proportion of liver‐ or substance use‐related deaths was over twice as high in those reporting heavy drinking, compared with no, low or moderate drinking.

Reasons for not drinking, or stopping drinking alcohol, may differ between PWH with and without HCV: PWH with HCV may stop or reduce their alcohol use because of knowledge of their HCV diagnosis^24^ or HCV‐related illness, while those without HCV, may be more likely to have stopped drinking due to other comorbidities, potentially related to previous alcohol use. Abstaining from alcohol is often due to religious or cultural reasons. Other behaviours may differ between PWH with and without HCV: in particular there is a higher prevalence of active IDU among PWH with than without HCV: this is associated with higher mortality due to overdose, and worse HIV‐related outcomes.^25^ It is possible that PWH with no history of heavy alcohol use were prioritised for DAAs early in the DAA‐era, resulting in reduced HCV‐mortality. Finally, higher mortality among the comparator low/moderate alcohol use group in those with HCV than without HCV may contribute to the effect modification.

### 4.1 Comparison with other literature

Our study is, to our knowledge, the first to demonstrate modification by HCV of the association of alcohol use with all‐cause mortality among PWH. Higher mortality among PWH with high alcohol use has been reported among those with and without HCV,^20^, ^26^, ^27^, ^28^ and binge‐drinking is associated with higher mortality.^29^ Alcohol use in PWH with HCV may exacerbate liver damage arising from prior long‐term HCV infection by causing oxidative stress and promoting fibrosis, thereby accelerating progression to cirrhosis.^30^ High alcohol use can lead to reduced adherence to medications, while alcohol consumption is itself associated with other comorbidities, such as cardiovascular disease.^19^, ^31^ Higher mortality among PWH who do not drink alcohol than among light/moderate drinkers may be partially explained by their prior drinking history.^27^ Differing proportions of abstaining from alcohol have been reported across HCV/HIV/IDU subgroups, with abstention from alcohol more common among people without a history of IDU that had HCV than those without HCV,^32^ although we did not see this in our study. The ART‐CC previously found that 62% of PWH with HCV had histories of IDU, compared to only 2% of PWH without HCV.^3^ Nonetheless, excess mortality in PWH with HCV was only partly explained by IDU and even after adjusting for IDU and other important confounding factors, PWH with HCV had a two times higher all‐cause mortality and 7.5 times higher liver‐related mortality than those without HCV.^3^ Several extra‐hepatic co‐morbidities (e.g. cardiovascular disease and non‐AIDS cancers) are observed more often in PWH with than without HCV.^33^, ^34^

### 4.2 Strengths and limitations

This analysis was based on multiple cohorts of PWH spanning many countries across the US and Europe. However, there were several limitations. Firstly, individuals' alcohol use will vary over time, but our analyses were based on a single report of alcohol use for each individual, so we may miss changes in alcohol use. We performed a sensitivity analysis to investigate the effect of restricting the window of alcohol use values to between 6 months before and 3 months after ART start and found similar results. Second, alcohol data were collected in different ways by different cohorts, and detailed analyses were required to harmonise these data (see supplementary materials), which may have resulted in the use of broad categories that do not capture enough variation in alcohol use. Self‐reported alcohol use may be affected by recall bias and perceived desirability of lower alcohol use, so the percentage of people in the highest alcohol use category may be an underestimate. Equally, the percentage of people self‐reporting as not drinking alcohol may be an overestimate. However, we have no evidence to suggest that any misreporting of drinking was not equivalent among both PWH with and without HCV, so our overall conclusions regarding the interaction between alcohol use and HCV status would likely remain unchanged. Due to a lack of data, we were unable to adjust our regression models for potential variables that may explain non‐drinking such as religious or cultural reasons, and previous diagnoses of alcohol use disorders. The database close date for most cohorts (~2019) occurred 5 years after the start of the DAA‐era of HCV treatment, so some HCV treatment information is for interferon‐based treatments, which are now rarely used in high‐income settings. Finally, when HCV RNA status was unavailable at ART start we used antibody status to define HCV‐infection, which may mean that we included some individuals in the HCV group who had previously spontaneously cleared their infections. However, our subsequent time‐updating of HCV status was performed only using HCV RNA testing data.

### 4.3 Implications

Both alcohol use and HCV are major causes of morbidity and mortality among PWH, whose importance is increasing as the rates of death due to AIDS decrease and the population of PWH on ART ages with corresponding increases in age‐related comorbidities and consumption of medications for these.^35^ The relative consequences of high versus moderate alcohol use are similar in PWH with and without HCV, which implies the excess mortality risk is higher among those with HCV, as their underlying mortality risk is greater. This suggests that interventions to reduce high alcohol use among PWH with HCV may lead to lower mortality. Further research is required to understand alcohol use patterns among PWH with and without HCV, and to develop appropriate interventions to reduce alcohol use.

## 5 CONCLUSIONS

We found differing associations between alcohol use and mortality among PWH with and without HCV. Whether mortality patterns among PWH who have been cured of or cleared HCV follow the patterns of PWH with or without HCV will require further analyses with longer follow‐up during the DAA era.

## FUNDING INFORMATION

The ART‐CC is funded by the US National Institute on Alcohol Abuse and Alcoholism (U01‐AA026209). JACS is funded by National Institute for Health Research Senior Investigator award NF‐SI‐0611‐10168. AT is funded by the Wellcome Trust under a Sir Henry Wellcome Postdoctoral Fellowship (222770/Z/21/Z). Funding for the individual ART‐CC cohorts included in this analysis was from Alberta Health, Gilead, ANRS‐MIE (Maladies Infectieuses Emergentes), the French Ministry of Health, the Austrian Agency for Health and Food Safety (AGES), Stichting HIV Monitoring, the Dutch Ministry of Health, Welfare and Sport through the Centre for Infectious Disease Control of the National Institute for Public Health and the Environment, the TP‐HIV by the German Centre for Infection Research (DZIF) (NCT02149004), the Instituto de Salud Carlos III through the Red Temática de Investigación Cooperativa en Sida (RD06/006, RD12/0017/0018 and RD16/0002/0006) as part of the Plan Nacional I + D + i and co‐financed by ISCIII‐Subdirección General de Evaluación and the Fondo Europeo de Desarrollo Regional (FEDER), ViiV Healthcare, Preben og Anna Simonsens Fond, Institut National de la Santé et de la Recherche Médicale (INSERM), BMS, Janssen, MSD, the US National Institute on Alcohol Abuse and Alcoholism (U01‐AA026230), the Spanish Ministry of Health, the Swiss National Science Foundation (grant 33CS30_134277), CFAR Network of Integrated Clinical Systems (1R24 AI067039‐1, P30‐AI‐027757), the US Department of Veterans Affairs, the US National Institute on Alcohol Abuse and Alcoholism (U01‐AA026224, U01‐AA026209, U24‐AA020794), the VHA Office of Research and Development, US National Institute of Allergy and Infectious Diseases (Center for AIDS Research: P30 AI110527).

## CONFLICT OF INTEREST STATEMENT

MJG has received honoraria in the last 3 years from ad hoc membership of national HIV advisory boards, Merck, Gilead, and ViiV. IJ has received teaching fees from ViiV, fees for evaluating scientific projects and participating in expert panels from Gilead, and fees for statistical analyses from GESIDA. NO's institution has received funding from the Preben og Anne Simonsens Fond. GT has received research grant funding independent of the current work from Gilead, EU, UCL, Novo Nordisk and National Funds, all paid to her institution. RZ has not received honoriara in the last 3 years. AR reports support to his institution for advisory boards and/or travel grants from MSD, Gilead Sciences, Pfizer and Abbvie, and an investigator‐initiated trial (IIT) grant from Gilead Sciences. All remuneration went to his home institution and not to AR personally, and all remuneration was provided outside the submitted work. FB has received travel grants and honoraria from ViiV Healthcare, Gilead, ViiV, Janssen, and MSD, and support for attending meetings from Gilead, Janssen, MSD, and ViiV Healthcare. GB has received consulting fee from MedIQ, and payments and honoraria from the University of Kentucky and StateServ, whilst GB's institution has received funding from Merck, Eli Lily, Kaiser Permanente, and Amgen. HC has received research grant funding from ViiV, NIH, and AHRQ paid to their institution, and sits on the NIH Office of AIDS Research Advisory Council. RT has received grant funding from Gilead unrelated to this work. JB has received honoraria for advice or public speaking from GILEAD, MSD, JANSSEN and ViiV Healthcare; and grants from GILEAD, MSD, and ViiV Healthcare. CW has received consulting fees from Gilead Sciences, ViiV Healthcare, MSD, Janssen and Roche–none related to the content of this manuscript. MH has received honoraria for participating to adboards from ViiV Healthcare and Gilead, and support for attending meetings from Gilead, Abbvie, MSD, and ViiV Healthcare. Through PR's institution, PR has received independent scientific grant support from Gilead Sciences, Merck & Co and ViiV Healthcare, and has served on scientific advisory boards for Gilead Sciences, ViiV Healthcare, and Merck & Co, for which honoraria were all paid to his institution–none related to the content of this manuscript. AT, SI, AB, SG, CTR, DDS, MJS, JG, SA, AdM, KM, JACS and LW report no conflicts of interest.

## Supporting information


**Table S1:** Unadjusted mortality hazard ratios for alcohol use categories, stratified by calendar year period and HCV status (negative, positive), splitting follow‐up time by HCV status.
**Table S2:** Adjusted* mortality hazard ratios for alcohol use categories post‐2014 for PWH that had ever had HCV**.

## Data Availability

Due to the data sharing agreements between individual cohorts and ART‐CC, the data collected for this study cannot be shared. Data are owned by the individual cohorts and those wishing to access these data should contact the individual cohorts.
