# Supplementary material for: Contribution of alcohol use in HIV/hepatitis C virus co‐infection to all‐cause and cause‐specific mortality: A collaboration of cohort studies
Source: J Viral Hepat. 2023 Jun 20;30(9):775–86. doi: 10.1111/jvh.13863 (PMC10526649; doi:10.1111/jvh.13863)
Supplement: Supplementary file 1 — Table S1: Unadjusted mortality hazard ratios for alcohol use categories, stratified by calendar year period and HCV status (negative, positive), splitting follow‐up time by HCV status. Table S2: Adjusted* mortality hazard ratios for alcohol use categories post‐2014 for PWH that had ever had HCV**. [file JVH-30-775-s001.docx]

**Supplementary materials**

**Harmonizing alcohol data, taken from S.M. Ingle et al; Harmonisation of data on alcohol use across a multi-national HIV cohort collaboration (unpublished).**

*AUDIT-C score*

Several cohorts (‘AUDIT-C cohorts’) collected alcohol data using the AUDIT-C tool: a three-item questionnaire which returns a score from 0-12 where increasing score means higher risk alcohol use.

*Grams of alcohol per day*

For the cohorts which did not collect alcohol data using the AUDIT-C tool, we calculated alcohol use as grams of alcohol per day. Several non-AUDIT-C cohorts recorded the actual number of drinks/units per week/day. The Italian (standard drink=12g) ICONA cohort recorded number of glasses of wine/bottles of beer/shots per day. The Canadian (standard drink=13.6g) Alberta cohort recorded a categorical variable: Level I (<9 drinks per week for women, <14 drinks per week for men), Level II (≥9 drinks per week for women, ≥14 drinks per week for men), None. We took the mid-point for Level I and the lowest value for Level II. The German (standard drink=12g) For those who drink, we took the mid-point of the category to define grams of alcohol. The French (standard drink=10g) FHDH cohort recorded a categorical variable: 0, <4 glasses per day, 4-8 glasses per day, >8 glasses per day, non-drinker, ex-drinker. For those who drink, we took the mid-point of the category to define grams of alcohol.

To allow comparisons between the AUDIT-C and non-AUDIT-C cohorts, we used AUDIT-C items one (frequency of drinking) and two (number of drinks on a typical day of drinking) to calculate grams of alcohol per day (1 drink=14g, except Switzerland 10g) for the AUDIT-C cohorts.

Due to the available mid-point values, alcohol use was categorised as: non-drinker (0 grams per day), moderate drinker (0.1-20 grams per day) and heavy drinker (20 grams or more per day).

### **Contribution of alcohol use in HIV/hepatitis C virus co-infection to all-cause and cause-specific mortality: a collaboration of cohort studies**

**SUPPLEMENTARY MATERIALS**

**Supplementary table 1:** Unadjusted mortality hazard ratios for alcohol use categories, stratified by calendar year period and HCV status (negative, positive), splitting follow-up time by HCV status.

|  | **All patients** | | **HCV negative** | | **HCV positive** | |  |
| --- | --- | --- | --- | --- | --- | --- | --- |
| **Alcohol use (grams per day)** | **Mortality rate per 1000 person-years (95% CI)** | **HR (95% CI)** | **Mortality rate per 1000 person-years (95% CI)** | **HR (95% CI)** | **Mortality rate per 1000 person-years (95% CI)** | **HR (95%CI)** | **Interaction p-value** |
| **Follow-up between 2001-2017 (N=58769)** | | | | | | | |
| Unadjusted |  |  |  |  |  |  | <0.001 |
| 0.0g | 7.3 (7.0-7.7) | 1.35 (1.25-1.46) | 6.1 (5.8-6.4) | 1.35 (1.24-1.47) | 21.5 (19.5-23.7) | 1.10 (0.95-1.28) |  |
| 0.1-20.0g | 6.6 (6.3-7.0) | 1 | 5.5 (5.2-5.9) | 1 | 21.4 (19.0-24.0) | 1 |  |
| >20.0g | 12.8 (11.8-13.9) | 2.17 (1.94-2.41) | 10.5 (9.5-10.7) | 2.06 (1.82-2.33) | 28.6 (24.2-33.7) | 1.74 (1.42-2.13) |  |
| **Follow-up between 2001-2013 (N=46174)** | | | | | | | |
| Unadjusted |  |  |  |  |  |  | <0.001 |
| 0.0g | 6.4 (5.9-6.8) | 1.32 (1.18-1.48) | 5.0 (4.6-5.4) | 1.34 (1.18-1.53) | 19.2 (17.0-21.8) | 1.03 (0.85-1.26) |  |
| 0.1-20.0g | 5.8 (5.3-6.2) | 1 | 4.5 (4.1-4.9) | 1 | 19.4 (16.7-22.5) | 1 |  |
| >20.0g | 11.8 (10.4-13.3) | 2.25 (1.92-2.63) | 9.2 (7.9-10.7) | 2.14 (1.77-2.58) | 26.3 (21.2-32.6) | 1.73 (1.32-2.26) |  |
| **Follow-up between 2014-2017 (N=54884)** | | | | | | | |
| Unadjusted |  |  |  |  |  |  | <0.001 |
| 0.0g | 8.4 (7.9-9.0) | 1.41 (1.27-1.56) | 7.3 (6.9-7.9) | 1.40 (1.25-1.56) | 26.1 (22.4-30.3) | 1.21 (0.96-1.54) |  |
| 0.1-20.0g | 7.6 (7.0-8.1) | 1 | 6.6 (6.1-7.2) | 1 | 25.0 (20.9-29.9) | 1 |  |
| >20.0g | 13.9 (12.3-15.7) | 2.11 (1.82-2.45) | 12.0 (10.4-13.7) | 2.03 (1.72-2.40) | 32.7 (25.2-42.4) | 1.68 (1.22-2.31) |  |

**Supplementary table 2:** Adjusted* mortality hazard ratios for alcohol use categories post-2014 for PWH that had ever had HCV**.

| **Adjusted mortality hazard ratios (95% confidence interval) (interaction term p-value 0.0063)** | | | |
| --- | --- | --- | --- |
| **Alcohol use (grams per day)** | **All observations (n=8973)** | **Previously HCV-positive (n=2813)** | **Currently HCV-positive (n=4807)** |
| 0.0g | 1.08 (0.84-1.38) | 1.51 (0.91-2.51) | 0.98 (0.74-1.29) |
| 0.1-20.0g | 1 | 1 | 1 |
| >20.0g | 1.32 (0.93-1.88) | 2.32 (1.16-4.63) | 1.13 (0.76-1.69) |

*The covariates included were HIV acquisition group, female, prior AIDS status, age, CD4 count cells/μL, and log HIV-1 RNA copies/mL.

**Multiple time periods (observations) are included per patient, split by time of HCV infection and cure/spontaneous clearances. There are 333 deaths among 4576 subjects, with 8973 observations.
